# Response to Farrokhi et al.’s statistical comments on ‘no seasonal variation in physical activity of Han Chinese living in Beijing’

**DOI:** 10.1186/s12966-017-0603-y

**Published:** 2017-11-03

**Authors:** G. Wang, J. R. Speakman

**Affiliations:** 1 State Key Laboratory of Molecular Developmental Biology, Institute of Genetics and Developmental Biology, Chinese Academy of Sciences, Beijing, 100101 China; 2 University of Chinese Academy of Sciences, Beijing, 100049 China; 3 Institute of Biological and Environmental Sciences, University of Aberdeen, Aberdeen, AB24 2TZ UK

**Fig. 1:**
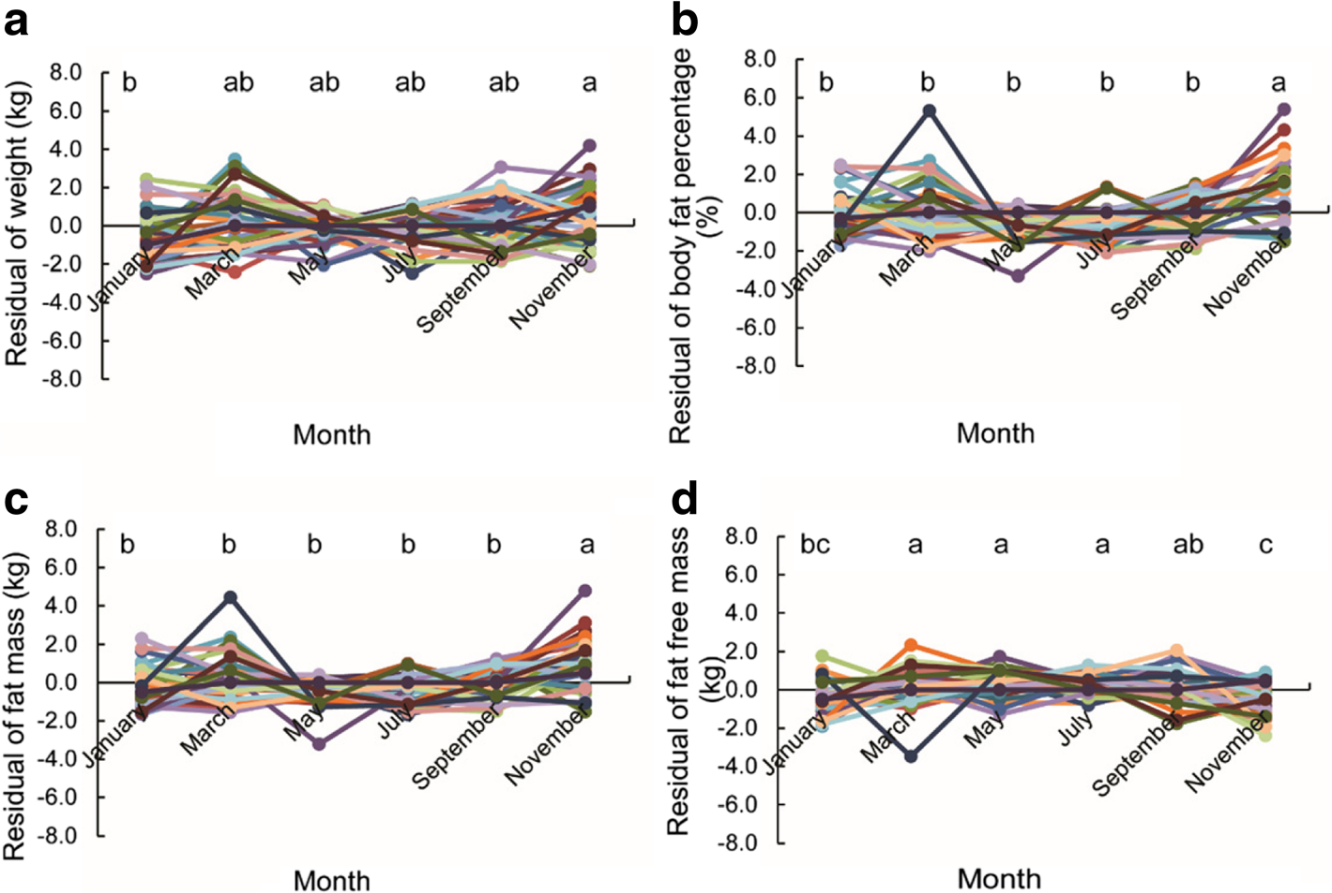
Temporal changes in body composition. **a** is the temporal changes in body weight. Each point represents residual of the actual value minus the mean value for the same individual. **b** represents the temporal changes in body fat percentage. **c** and **d** show the temporal changes in fat mass and fat-free mass. Means that do not share a letter are significantly different

Dear Editor,

We have previously shown that physical activity patterns of Han Chinese adults living in Beijing were relatively invariant over the course of a single year [1], despite enormous differences in ambient temperature over the annual cycle. In addition we also indicated that BMI and body composition of the same subjects was also not significantly changed over the course of the year long measurements. We are grateful to Farrokhi et al. [2] for pointing out an error in our analysis in this latter section of the paper, and welcome the opportunity to amend this analysis in the light of their constructive comments. As noted by Farrokhi et al. [2] in our analysis of the effects of time on body composition parameters we used a simple one-way ANOVA that did not account for the fact the measurements were repeated in the same individuals and hence not independent. When we repeat the analysis as a General Linear Model including individual ID as a random factor, then it turns out that there are indeed significant temporal effects on the body composition measurements (Body weight: F_Month (5195)_ = 0.66, *p* = 0.024, F_ID (33,195)_ = 247.19, *p* < 0.001; BF%: F_Month (5195)_ = 11.49, *p* < 0.001, F_ID (33,195)_ = 229.50, *p* < 0.001; FM: F_Month (5195)_ = 9.15, *p* < 0.001, F_ID (33,195)_ = 159.63, *p* < 0.001; FFM: F_Month (5195)_ = 6.44, p < 0.001, F_ID (33,195)_ = 524.33, *p* < 0.001). These effects are illustrated in Fig. 1. The body weight in January was significantly lower than in November, but the other months did not differ significantly from each other. For body fat percentage and fat mass, the value in November was greater than in the other months. Fat-free mass was higher in the summer months compared to the winter (Fig. 1d).

We note from these revised analyses that for the body composition measurements we made the main effect was that the individuals were significantly fatter and heavier later in the study (November) compared with earlier (January). Because our measurements spanned only a single year we cannot distinguish whether this was a seasonal effect, or because the individuals were all getting older as the study progressed. In contrast fat-free mass did not progressively increase but was higher both at the start and end, but lower in the summer period in the middle. This may be a true seasonal effect, but additional years of study would be required to confirm that. Previous studies have shown adult individuals in the USA gain around 0.5 kg each year as they age [3] and the magnitude of this effect is consistent with the change we observed in weight and fatness. We emphasise that the other statistical analyses in the original paper concerning physical activity levels did account for repeated measurements, and these analyses, and the conclusions drawn from them, are unaffected by this error.
